# Supplementary material for: Electrocorticographic dissociation of alpha and beta rhythmic activity in the human sensorimotor system
Source: eLife. 2019 Oct 9;8:e48065. doi: 10.7554/eLife.48065 (PMC6785220; doi:10.7554/eLife.48065)
Supplement: Source code 1. [file elife-48065-code1.pdf]

```matlab
%%%%%%%%%%%%%%%%%%%%%%%%%%%%%%%%%%%%%%%%%%%%%%%%%%%%%%%%%%%%%%%%%%%%%
% MATLAB script for the extraction of rhythmic spectral features
% from the electrophysiological signal based on Irregular Resampling
% Auto-Spectral Analysis (IRASA, Wen & Liu, Brain Topogr. 2016)
%
% Ensure FieldTrip is correcty added to the MATLAB path:
%   addpath <path to fieldtrip home directory>
%   ft_defaults
%
% From Stolk et al., Electrocorticographic dissociation of alpha and
% beta rhythmic activity in the human sensorimotor system
%%%%%%%%%%%%%%%%%%%%%%%%%%%%%%%%%%%%%%%%%%%%%%%%%%%%%%%%%%%%%%%%%%%%%


% generate trials with a 15 Hz oscillation embedded in pink noise
t = (1:1000)/1000; % time axis
for rpt = 1:100
  % generate pink noise
  dspobj = dsp.ColoredNoise('Color', 'pink', ...
    'SamplesPerFrame', length(t));
  fn = dspobj()';

  % add a 15 Hz oscillation
  data.trial{1,rpt} = fn + cos(2*pi*15*t);
  data.time{1,rpt}  = t;
  data.label{1}     = 'chan';
  data.trialinfo(rpt,1) = rpt;
end

% partition the data into ten overlapping sub-segments
w = data.time{1}(end)-data.time{1}(1); % window length
cfg               = [];
cfg.length        = w*.9;
cfg.overlap       = 1-((w-cfg.length)/(10-1));
data_r = ft_redefinetrial(cfg, data);

% perform IRASA and regular spectral analysis
cfg               = [];
cfg.foilim        = [1 50];
cfg.taper         = 'hanning';
cfg.pad           = 'nextpow2';
cfg.keeptrials    = 'yes';
cfg.method        = 'irasa';
frac_r = ft_freqanalysis(cfg, data_r);
cfg.method        = 'mtmfft';
orig_r = ft_freqanalysis(cfg, data_r);

% average across the sub-segments
frac_s = {};
orig_s = {};
for rpt = unique(frac_r.trialinfo)'
  cfg               = [];
```

```
  cfg.trials        = find(frac_r.trialinfo==rpt);
  cfg.avgoverrpt    = 'yes';
  frac_s{end+1} = ft_selectdata(cfg, frac_r);
  orig_s{end+1} = ft_selectdata(cfg, orig_r);
end
frac_a = ft_appendfreq([], frac_s{:});
orig_a = ft_appendfreq([], orig_s{:});

% average across trials
cfg               = [];
cfg.trials        = 'all';
cfg.avgoverrpt    = 'yes';
frac = ft_selectdata(cfg, frac_a);
orig = ft_selectdata(cfg, orig_a);

% subtract the fractal component from the power spectrum
cfg               = [];
cfg.parameter     = 'powspctrm';
cfg.operation     = 'x2-x1';
osci = ft_math(cfg, frac, orig);

% plot the fractal component and the power spectrum
figure; plot(frac.freq, frac.powspctrm, ...
  'linewidth', 3, 'color', [0 0 0])
hold on; plot(orig.freq, orig.powspctrm, ...
  'linewidth', 3, 'color', [.6 .6 .6])

% plot the full-width half-maximum of the oscillatory component
f    = fit(osci.freq', osci.powspctrm', 'gauss1');
mean = f.b1;
std  = f.c1/sqrt(2)*2.3548;
fwhm = [mean-std/2 mean+std/2];
yl   = get(gca, 'YLim');
p = patch([fwhm flip(fwhm)], [yl(1) yl(1) yl(2) yl(2)], [1 1 1]);
uistack(p, 'bottom');
legend('FWHM oscillation', 'Fractal component', 'Power spectrum');
xlabel('Frequency'); ylabel('Power');
set(gca, 'YLim', yl);
```

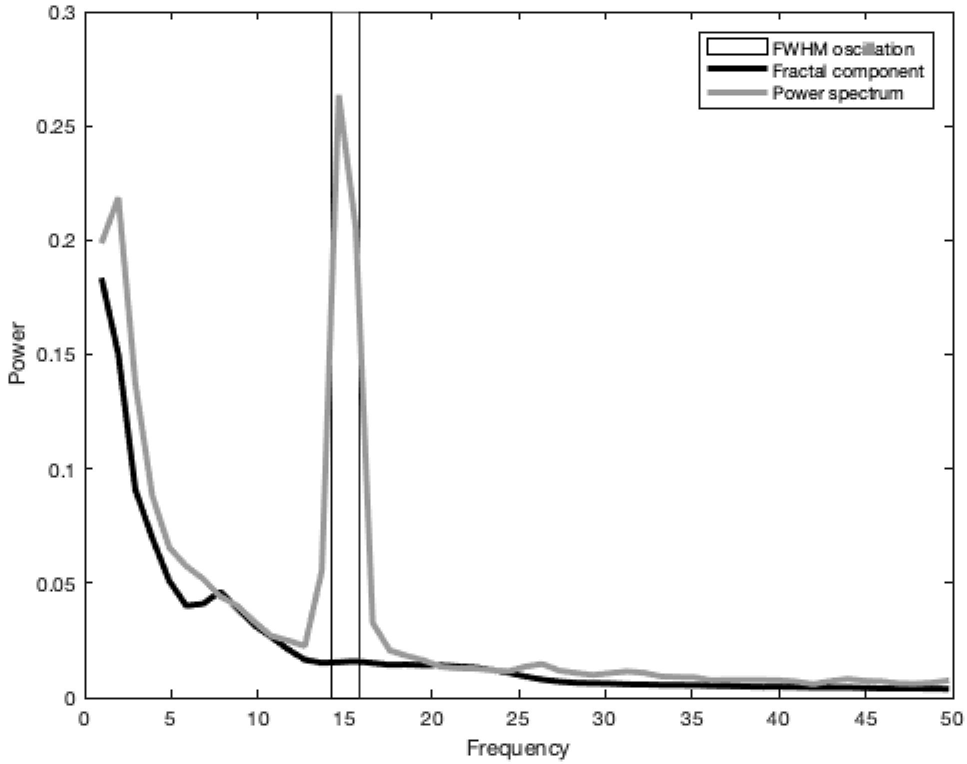

Published with MATLAB® R2017b
